# Cre‐driver lines used for genetic fate mapping of neural crest cells in the mouse: An overview

**DOI:** 10.1002/dvg.23105

**Published:** 2018-04-19

**Authors:** Julien Debbache, Vadims Parfejevs, Lukas Sommer

**Affiliations:** ^1^ Stem Cell Biology, Institute of Anatomy, University of Zurich Zurich CH‐8057 Switzerland

**Keywords:** Cre/LoxP system, lineage tracing, neural crest

## Abstract

The neural crest is one of the embryonic structures with the broadest developmental potential in vertebrates. Morphologically, neural crest cells emerge during neurulation in the dorsal folds of the neural tube before undergoing an epithelial‐to‐mesenchymal transition (EMT), delaminating from the neural tube, and migrating to multiple sites in the growing embryo. Neural crest cells generate cell types as diverse as peripheral neurons and glia, melanocytes, and so‐called mesectodermal derivatives that include craniofacial bone and cartilage and smooth muscle cells in cardiovascular structures. In mice, the fate of neural crest cells has been determined mainly by means of transgenesis and genome editing technologies. The most frequently used method relies on the *Cre*‐*loxP* system, in which expression of Cre‐recombinase in neural crest cells or their derivatives genetically enables the expression of a Cre‐reporter allele, thus permanently marking neural crest‐derived cells. Here, we provide an overview of the Cre‐driver lines used in the field and discuss to what extent these lines allow precise neural crest stage and lineage‐specific fate mapping.

## 1 A BRIEF SYNOPSIS OF METHODS USED FOR NEURAL CREST CELL FATE MAPPING

The astonishing variety of neural crest derivatives has long been recognized, mainly through the pioneering work by Le Douarin and co‐workers who used interspecies transplantation in avian embryos to determine normal fates as well as the developmental potential of neural crest cell populations from different axial levels of the embryo (Bronner & Simões‐Costa, 2016; Le Douarin & Dupin, 2003). Together with assays involving dye labeling and retroviral infection of neural crest cells, this approach led to the establishment of comprehensive fate maps and revealed that certain neural crest derivatives (such as peripheral glia, sensory neurons, and melanocytes) are produced from all axial levels, whereas other neural crest cell lineages (such as cartilage, bone, smooth muscle, connective tissue, endocrine cells as well as, for instance, neurons and glia from the enteric and parasympathetic nervous system) originate from discrete levels along the neuraxis.

Given the limited accessibility of mammalian embryos, lineage‐tracing experiments as performed in avian embryos have not been widely used for fate mapping of mammalian neural crest cells. Rather, various genetic tools have been established that allow noninvasive and long‐term tracing of neural crest cells in mice in vivo (Zurkirchen & Sommer, 2017). By far the most frequently used approach in the field is *Cre*‐*loxP*‐based conditional genetic recombination that, when combined with a Cre‐reporter line, results in inheritable and irreversible expression of a marker gene in Cre‐recombinase‐expressing cells and in all of their progeny (Woodworth, Girskis, & Walsh, 2017). Furthermore, inducible forms of Cre‐recombinase have been applied to perform stage‐dependent fate mapping of neural crest cells and their derivatives or fate mapping at low recombination density for in vivo single cell tracing (Baggiolini et al., 2015; Kaucka et al., 2016). Apart from confirming in mice many of the findings obtained by fate mapping of avian neural crest cells, genetic lineage tracing of murine neural crest cells led, for instance, to the identification of minor neural crest‐derived cell populations present in tissues of nonneural crest origin, to the establishment of novel lineage trees (revealing, in particular, the broad developmental potential of peripheral glial cells), and to the demonstration of in vivo multipotency of single premigratory and migratory neural crest cells. While these studies have recently been covered elsewhere (Petersen & Adameyko, 2017; Zurkirchen & Sommer, 2017), in the present review we aim to focus on the tool set used in the field, summarizing the findings made with and discussing specific properties of various Cre lines that have been utilized to trace neural crest cells.

## 2 TRACING OF PREMIGRATORY AND MIGRATORY NEURAL CREST CELLS USING NONINDUCIBLE CRE‐DRIVER LINES

A well‐established Cre‐driver line for neural crest lineage tracing is *Wnt1‐Cre* (Danielian, Muccino, Rowitch, Michael, & McMahon, 1998) (Table 1). This transgenic mouse line expresses Cre initially in the midbrain and, after closure of the neural tube, in the midlines of the midbrain and the caudal diencephalon, in the midbrain–hindbrain junction, and in the dorsal spinal cord, where it recombines premigratory neural crest cells. By crossing *Wnt1‐Cre* mice with the *ROSA26 (R26R)* Cre‐reporter line (that drives β‐galactosidase expression upon Cre‐mediated recombination) (Soriano, 1999), it was shown that *Wnt1‐Cre* is a highly efficient Cre‐driver line, resulting in recombination of approximately 96% of all migratory neural crest cells (Hari et al., 2012). Because Wnt1 is not expressed in migratory neural crest cells and Wnt activity rapidly decreases in neural crest cells after their delamination from the neural tube (Kléber et al., 2005; Rabadán et al., 2016; Zervas, Millet, Ahn, & Joyner, 2004), it can be assumed that most neural crest cells are very efficiently targeted by *Wnt1‐Cre* before or at the time of their delamination. Intriguingly, however, despite the early activity of *Wnt1‐Cre* in the dorsal neural tube, recombination apparently occurs too late to allow investigation of mechanisms regulating epithelial‐to‐mesenchymal transition (EMT) or delamination of neural crest cells. Indeed, *Wnt1‐Cre‐*mediated ablation of signaling pathways shown in other animal models to be crucial for neural crest EMT, such as canonical Wnt signaling and signaling by TGFβ superfamily factors, did not affect early stages of neural crest development (Brault et al., 2001; Büchmann‐Møller et al., 2009; Hari et al., 2002; Jia et al., 2007). In contrast, the differentiation potential of neural crest cells along all axial levels could readily be monitored using *Wnt1‐Cre*, both during development and at postnatal stages (Chai et al., 2000; Jiang, Rowitch, Soriano, McMahon, & Sucov, 2000; Zurkirchen & Sommer, 2017). A potential caveat of this line is, however, that, at least in the midbrain, Wnt1 is ectopically expressed from the *Wnt1‐Cre* transgene, which could lead to ectopic activation of canonical Wnt signaling (Lewis, Vasudevan, O'neill, Soriano, & Bush, 2013). Although it is not known whether such ectopic Wnt1 expression also affects the neural crest, the use of a new driver line termed *Wnt1‐Cre2* should be considered (Lewis et al., 2013). In fact, in studies addressing the role of fibronectin in cardiac neural crest development, considerable phenotypic variances have been reported upon *Wnt1‐Cre* vs. *Wnt1‐Cre2*‐mediated recombination, although this could have been due to differences between the two transgenic lines other than aberrant Wnt1 expression (Wang & Astrof, 2016).

**Table 1:** List of publicly available neural crest drivers

Premigratory neural crest drivers		
*Short name*	Official Name	Original study
*P3Pro‐Cre*	Tg(Pax3‐cre)1Joe	Li et al. (2000)
*Wnt1‐Cre*	H2afv^Tg(Wnt1‐cre)11Rth^	Danielian et al. (1998)
*Wnt1‐Cre2*	E2f1^Tg(Wnt1‐cre)2Sor^	Lewis et al. (2013)
*Wnt1‐CreER*	Tg(Wnt1‐cre/ERT)1Alj	Zervas et al. (2004)
*Wnt1‐Flpe*	Tg(Wnt1‐FLP1)1Dym	Dymecki et al. (1998)
*Wnt1‐FlpeER^T2^*	Tg(Wnt1‐flpe/ERT2)9455Dym	Hunter et al. (2005)

Migratory neural crest drivers		
*Short name*	Official name	Original study
*AP2α‐IRESCre*	*Tfap2a^tm1(cre)Moon^*	Macatee et al. (2003)
*Ht‐PA‐Cre*	Tg(PLAT‐cre)116Sdu	Pietri et al. (2003)
*Mef2c‐F10N‐Cre*	Tg(Mef2c‐cre)5Patr	Aoto et al. (2015)
*P0‐Cre*	Tg(Mpz‐cre)94Imeg	Yamauchi et al. (1999)
*Plp‐CreER^T^*	Tg(Plp1‐cre/ERT)3Pop/J	Doerflinger et al. (2003)
*Plp‐CreER^T2^*	Tg(Plp1‐cre/ERT2)1Ueli	Leone et al. (2003)
*Sox10‐Cre*	Tg(Sox10‐cre)1Wdr	Matsuoka et al. (2005)
*Sox10ER^T2^CreER^T2^* (SECE)	Tg(Sox10‐ERT2/cre/ERT2)17Sor	He and Soriano (2015)
*Sox10‐iCreER^T2^*	Tg(Sox10‐icre/ERT2)1Ldim	Simon et al. (2012)
*Sox10‐iCreER^T2^*	Tg(Sox10‐icre/ERT2)26Vpa	Laranjeira et al. (2011)
*Sox10‐iCreER^T2^*	Tg(Sox10‐icre/ERT2)388Wdr	McKenzie et al. (2014)
*TEC1*	Tg(Tyr‐cre)1Gfk	Tonks et al. (2003)

Apart from the *Cre/loxP* system, another site‐specific recombination system has also been established to trace the fate of neural crest cells. To this end, two transgenic mouse lines (termed *Wnt1‐Flpe* mice) were independently generated that express Flp recombinase from the *Wnt1* promoter (Dymecki & Tomasiewicz, 1998; Hatzistergos et al., 2015). Although the recombination efficiency and the extent of neural crest lineages traceable by these lines have not been described in detail, these lines were instrumental to perform intersectional lineage tracing of cells that concurrently express two distinct promoters. When combined with either the *RC::FrePe* (Engleka et al., 2012) or *RC::Fela* (Jensen et al., 2008) dual reporter alleles (which report dual Flp and Cre recombination), a fraction of *cKit‐CreER^T2^*‐traced cardiac progenitors was shown to derive from the cardiac neural crest (traced by *Wnt1‐Flpe*) (Hatzistergos et al., 2015). Likewise, intersectional fate‐mapping with the *RC::FrePe* allele was used to demonstrate that Isl1 is not an exclusive marker for second heart field cardiac progenitors, as previously suggested, but also marks a subpopulation of cardiac neural crest cells (Engleka et al., 2012).

Another mouse line expressing Cre in the dorsal neural tube and premigratory neural crest is *P3Pro‐Cre*, in which Cre expression is driven from a *Pax3* promoter fragment (Li, Chen, & Epstein, 2000). Although *Pax3* is expressed in the neural plate border before bona fide neural crest specification (Bronner & Simões‐Costa, 2016), Cre‐mediated conditional inactivation of pathways controlling EMT/delamination did not affect neural crest cell production and early migration in *P3Pro‐Cre* embryos (Buchmann‐Moller and Sommer, unpublished). Thus, we are not aware of a Cre‐driver line suitable for the study of early events in neural crest development, including neural crest specification, EMT, and delamination. Fate mapping experiments with *P3Pro‐Cre* have demonstrated efficient labeling of postmigratory neural crest derivatives, such as the enteric nervous system, the mesenchyme in pharyngeal arches, and cardiovascular structures. In contrast to the *Wnt1‐Cre* line, however, *P3Pro‐Cre*‐mediated recombination appears to be less specific for neural crest lineage tracing as it also marks noncrest neuroepithelial cells and several mesodermal tissues, including cartilaginous portions of the ribs and a large part of the skeletal musculature (Jarad & Miner, 2009; Li et al., 2000; Liu et al., 2006; Lang et al. 2000).

Several Cre‐driver lines have been generated that, unlike the *Wnt1‐Cre* or *P3Pro‐Cre* lines, express Cre‐recombinase in neural crest cells not before they undergo an EMT in the dorsal neural tube, but only as the cells begin to migrate. For instance, transgenic *Ht‐PA‐Cre* mice express Cre under the control of a human tissue plasminogen activator (Ht‐PA) promoter fragment specifically in migratory neural crest cells (Pietri, Eder, Blanche, Thiery, & Dufour, 2003). A detailed comparison with *Wnt1‐Cre/R26R* mice revealed very efficient labeling of neural crest derivatives by *Ht‐PA‐Cre*, including neuronal, glial, melanocytic, and mesenchymal cell populations during development and in adult structures (Pietri et al., 2003; Wong et al., 2006). *Ht‐PA‐Cre/R26R* mice were also reported to label a fraction of nonneural epithelial cells lateral to cranial neural folds that were suggested to contribute to mesectodermal structures in the head (Breau, Pietri, Stemmler, Thiery, & Weston, 2008).

Additional Cre‐driver lines mostly used to study cranial and cardiac neural crest development are the transgenic line *Mef2c‐F10N‐Cre* (Aoto et al., 2015) and the *AP2*α‐*IRESCre* line generated by knock‐in of an IRESCre cassette into the 3′ untranslated region of the AP2α transcription factor locus (Macatee et al., 2003). A detailed analysis of *Mef2c‐F10N*‐*Cre*/*R26R* embryos at different stages confirmed the neural crest origin of various neural and nonneural structures and emphasized the neural crest origin of olfactory ensheathing glial cells, cells in the meninges surrounding the forebrain, and cells of the choroid plexus. Apart from marking migratory neural crest cells only after delamination from the neural tube, *Mef2c‐F10N*‐*Cre*/*R26R* presented a neural crest fate map highly similar to the one obtained with the *Wnt1*‐Cre line, with the exception of some differences apparent in the calvarial bones (Aoto et al., 2015).

Tyrosinase (Tyr) is a key enzyme of the melanin biosynthetic pathway and, accordingly, a transgenic mouse line, in which *Tyr* enhancer and promoter elements drive Cre expression, was found to mark the melanocytic lineage, but not other neural crest derivatives (Delmas, Martinozzi, Bourgeois, Holzenberger, & Larue, 2003). In contrast, an independently produced *Tyr‐Cre* line termed *TEC1* also marks neural crest lineages other than melanoblasts, such as craniofacial structures, dorsal root ganglia (DRG), and sympathetic cephalic ganglia, albeit at a seemingly low recombination efficiency and at a relatively late stage in neural crest development (embryonic day (E) 10.5 onwards) (Tonks et al., 2003). Whether this reflects activity of *tyrosinase* transcriptional elements in a subset of undifferentiated migratory neural crest cells or aberrant transgene expression remains to be determined.

Although P0 was originally identified as a marker of the peripheral glial lineage, the expression of *P0‐Cre* in transgenic mice was not lineage restricted, but detected already in migratory neural crest cells (Yamauchi et al., 1999). Consequently, *P0‐Cre/R26R* mice displayed β‐galactosidase expression in multiple neural and nonneural tissues originating from the neural crest. However, at least in some structures such as the DRG, the recombination efficiency appeared to be considerably lower than the one achieved by *Wnt1‐Cre*‐mediated recombination. Furthermore, cranial neural crest cell populations are differentially marked in *Wnt1‐Cre* vs. *P0‐Cre* mice: *Wnt1‐Cre* preferentially recombines midbrain as opposed to hindbrain neural crest cells, while hindbrain neural crest cells are efficiently targeted by *P0‐Cre* (Chen et al., 2017). Intriguingly, fate‐mapping experiments by means of the *P0‐Cre* line also allowed the identification of neural crest‐derived cells in structures previously not known to harbor such cells. Specifically, Nagoshi and colleagues demonstrated that neural crest cells give rise to vascular endothelial and smooth muscle cells present in the adult bone marrow, which was later confirmed in *Wnt1‐Cre* mice (Nagoshi et al., 2008; Wislet‐Gendebien et al., 2012). Likewise, between 5 and 15% of all hormone‐producing cells in the anterior lobe of the pituitary turned out to originate from the neural crest based on *P0‐Cre*‐ lineage tracing (Ueharu et al., 2017).

**Figure 1:**
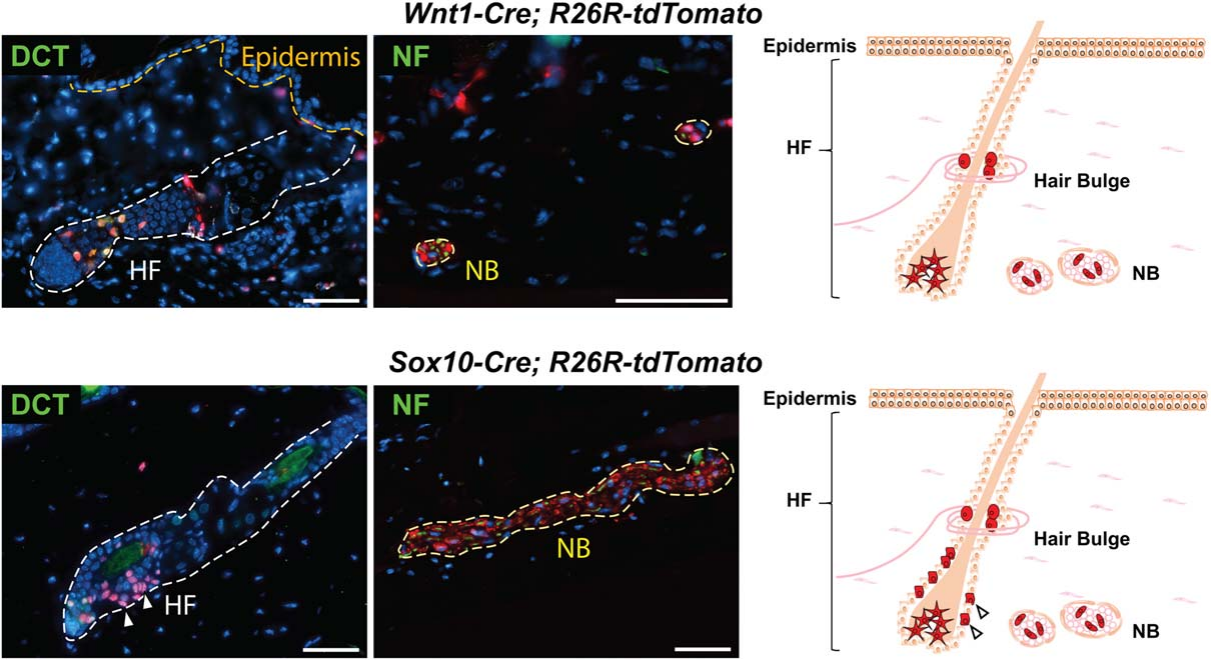
Adult skin structures traced by *Wnt1‐Cre* and *Sox10‐Cre*‐driven recombination. Immunolabeling of adult skin from mice, in which recombination of the Cre‐reporter allele *R26R‐tdTomato* was driven during neural crest development by *Wnt1‐Cre* (Danielian et al., 1998) and *Sox10‐Cre* (Matsuoka et al., 2005), respectively. Genetically recombined cells are labeled with tdTomato (red) in combination with selected lineage markers (green): DCT (melanocytes in hair follicles (HF)); NF (axons of nerves in nerve bundles (NB)). Aberrant tracing of some keratinocytes (white arrows) can be observed with *Sox10‐Cre*. Hoechst nuclei counterstaining, scale bars 50 µm. The schematics summarize the neural crest lineage‐specific and unspecific recombination events observed with these Cre‐driver lines. Star‐like cells: melanocytes; cells in hair bulges: glial cells and melanocyte stem cells; cells in NBs: Schwann cells

Yet another transgenic mouse line expressing Cre in neural crest cells only once they have engaged in migration is *Sox10‐Cre* (Hari et al., 2012; Matsuoka et al., 2005). Fate mapping in *Sox10‐Cre/R26R* embryos, combined with immunostaining for Cre protein, demonstrated targeting of approximately 78% of all Sox10‐positive neural crest cells after their emigration from the neural tube (Hari et al., 2012). Thanks to this high recombination efficiency it was possible to address stage‐specific functions of canonical Wnt signaling upon constitutive activation in premigratory neural crest (using *Wnt1‐Cre*) vs. migratory neural crest cells (using *Sox10‐Cre*). Furthermore, the *Sox10‐Cre* line was used together with the *Wnt1‐Cre* line to show an unexpected contribution of neural crest cells in the murine neck region to muscle connective tissue, cartilage and bone, including endochondral bones that were till then believed to exclusively originate from the mesoderm (Matsuoka et al., 2005). However, unlike *Wnt1‐Cre*, *Sox10‐Cre* activity leads to recombination of some adult structures that are not of neural crest origin, such as subpopulations of epithelial cells present in hair follicles (Figure 1).

## 3 NEURAL CREST LINEAGE TRACING USING INDUCIBLE CRE‐DRIVER LINES

The above‐described Cre lines allow *in vivo* fate mapping or functional analysis of a gene of interest only at the first time the Cre‐driving promoter is active. To temporally control Cre activity, inducible forms of Cre have been developed, for instance by fusion with a mutant ligand‐binding domain of the human estrogen receptor (CreER) that bind to the synthetic estrogen receptor ligand 4‐hydroxytamoxifen (4‐OHT) or tamoxifen (TM), but not to endogenous estradiol (Feil et al., 1996). Subsequently, a refined version with a higher TM sensitivity was constructed (termed CreER^T2^) (Feil, Wagner, Metzger, & Chambon, 1997). Although some mouse lines expressing inducible forms of Cre display leakiness, CreER‐ and CreER^T2^‐expressing lines have become very valuable tools to determine stage‐specific roles of genes of interest, to carry out lineage tracing experiments at different stages of neural crest development, or to perform clonal analyzes of neural crest cells *in vivo* by choosing conditions enabling low recombination frequencies (Zurkirchen & Sommer, 2017).

Because Wnt1 is only expressed in premigratory neural crest, but not once neural crest cells have emigrated from the neural tube, *Wnt1‐CreER* and *Wnt1‐FlpeER^T2^* mice (generated to study lineage relationships of cells at the mid/hindbrain boundary (Hunter, Awatramani, Farley, & Dymecki, 2005; Zervas et al., 2004)) (Table 1) are not suitable for neural crest lineage tracing at different time points. However, the *Wnt1‐CreER* line has been successfully used in conjunction with the multicolor Cre‐reporter allele *R26R‐Confetti* (Snippert et al., 2010) to show that premigratory *Wnt1*‐expressing neural crest cells are multipotent *in vivo* (Baggiolini et al., 2015). In this study, low dose TM treatment of pregnant mice led to low density recombination of the multicolor Cre‐reporter in neural crest cells homozygous for *R26R‐Confetti*, resulting in clones of cells expressing either nuclear green, cytoplasmic yellow, cytoplasmic red, membrane‐bound blue, or rare combinations thereof. Intriguingly, around 20% of all the clones derived from single *Wnt1‐CreER‐*traced neural crest cells not only contributed to multiple neural crest cell lineages, but also contained daughter cells in the dorsal neural tube. These data are consistent with the idea that at least some premigratory neural crest cells self‐renew in vivo.

The assumption that multipotent neural crest cells can self‐renew during a given time window was further supported by clonal analysis of migratory neural crest cells, traced shortly after emigration by means of a *Sox10‐iCreER^T2^* driver line (Baggiolini et al., 2015; Simon, Lickert, Götz, & Dimou, 2012). By using *R26R‐Confetti* as Cre‐reporter in *Sox10‐iCreER^T2^* mice treated with low TM doses, the vast majority of migratory neural crest cells were shown to maintain multipotency, and the *Sox10‐iCreER^T2^*‐expressing cells did not display any higher degree of fate restriction as compared to their premigratory counterparts (Baggiolini et al., 2015). The only structure labeled by *Wnt1‐CreER–* but not *Sox10‐iCreER^T2^*–lineage tracing was the dorsal neural tube, confirming that *Sox10‐iCreER^T2^* is not expressed in the premigratory neural crest, but only once the cells start to migrate. *R26R‐Confetti*‐based clonal analysis of neural crest cells has also been performed with another, independently produced *Sox10‐iCreER^T2^* line (Kaucka et al., 2016; Laranjeira et al., 2011). In this study, composition and behavior of ectomesenchymal cranial neural crest‐derived clones were monitored during early craniofacial development, revealing that cranial neural crest cell‐derived clones frequently comprise odontogenic, chondrogenic, osteogenic, and adipogenic cells.

Sox10 is not only expressed in migratory neural crest cells, but its expression is maintained throughout development and postnatally in the glial and melanocyte lineage, including in adult tissue (Bremer et al., 2011; Kuhlbrodt, Herbarth, Sock, Hermans‐Borgmeyer, & Wegner, 1998; Shakhova et al., 2012). Moreover, it marks cells with neural crest stem cell (NCSC) features that have been isolated from various adult neural crest‐derived structures (Shakhova & Sommer, 2010). Importantly, reporter expression in *Sox10‐iCreER^T2^* lines nicely matches the known Sox10 expression pattern during development and in the adult (Laranjeira et al., 2011; Simon et al., 2012). Thus, these *Sox10‐CreER^T2^* lines (Laranjeira et al., 2011; Simon et al., 2012) are ideally suited to monitor whether neural crest‐derived cells give rise to distinct cell populations at different time points or to assess stage‐specific roles of genes of interest. In fact, the *Sox10‐iCreER^T2^* line by Laranjeira and colleagues has been used to show that glia in the adult enteric nervous system are able to produce neurons upon injury (Laranjeira et al., 2011) or that cells in peripheral nerves can give rise to parasympathetic neurons, dental mesenchymal cells, or neuroendocrine chromaffin cells in the adrenal medulla (Dyachuk et al., 2014; Furlan et al., 2017; Kaukua et al., 2014). Such studies might also be possible with yet another independently generated *Sox10‐iCreER^T2^* line (McKenzie et al., 2014) or with a *Sox10ER^T2^CreER^T2^* line termed *SECE* (He & Soriano, 2015). Of note, while high dose TM treatment in *SECE/R26R* embryos resulted in labeling of multiple neural and nonneural neural crest derivatives, low dose application of TM was found to affect the reporter gene expression pattern, allowing the tracing specifically of cranial as opposed to trunk neural crest cells (He & Soriano, 2015).

Another genetic tool suitable for neural crest cell fate mapping at different developmental stages is a *Plp‐CreER^T2^* mouse line generated by Leone and colleagues (Leone et al., 2003). Plp is a glia‐specific marker, and the *Plp* gene regulatory elements used to drive *CreERT2* expression in these mice were reported to direct specific transgene expression in oligodendrocytes and Schwann cells. Accordingly, TM‐induced recombination during embryonic development and in adult mice led to very efficient labeling of peripheral glia in *PLP‐CreER^T2^/R26R* double transgenic animals (approximately 80% upon TM injection of adult mice) (Leone et al., 2003). Moreover, using this line, an unexpectedly broad developmental potential of peripheral glial cells was revealed, with *Plp‐CreER^T2^*‐traced cells along peripheral nerves giving rise to cell types as diverse as melanocytes, parasympathetic neurons, mesenchymal cells in teeth, and chromaffin cells of the adrenal medulla (Adameyko et al., 2009; Dyachuk et al., 2014; Furlan et al., 2017; Kaukua et al., 2014; Petersen & Adameyko, 2017). The glial origin of at least some melanocytes was also suggested by fate mapping experiments using another inducible *Plp‐Cre‐*driver line, *Plp‐CreER* (Doerflinger, Macklin, & Popko, 2003). Using this line, it was reported that melanocytes in hair follicles, but not in the interfollicular epidermis of the tail, originate from *Plp‐Cre*‐positive glial cells at E11.5 in the mouse (Deo, Huang, Fuchs, de Angelis, & Van Raamsdonk, 2013). The potential of peripheral glia to generate nonglial cell types was also demonstrated by alternative approaches, involving fate mapping with other lineage‐specific Cre‐driver lines or single cell RNA sequencing (Espinosa‐Medina et al., 2014; Furlan et al., 2017; Uesaka, Nagashimada, & Enomoto, 2015). The latter allowed generation of “pseudo‐time” lineage trajectories and revealed an intermediate cellular state in between the states defining Schwann cell precursors and differentiated nonglial cells, respectively (Furlan et al., 2017). Finally, the *Plp‐CreER^T2^* line (Leone et al., 2003) was instrumental to demonstrate by fate mapping that adult peripheral glia become activated upon skin wounding, detach from axons, and colonize the wound bed to support wound healing in a paracrine manner, without notable differentiation into other, nonglial cell types (Parfejevs et al., 2018).

**Figure 2:**
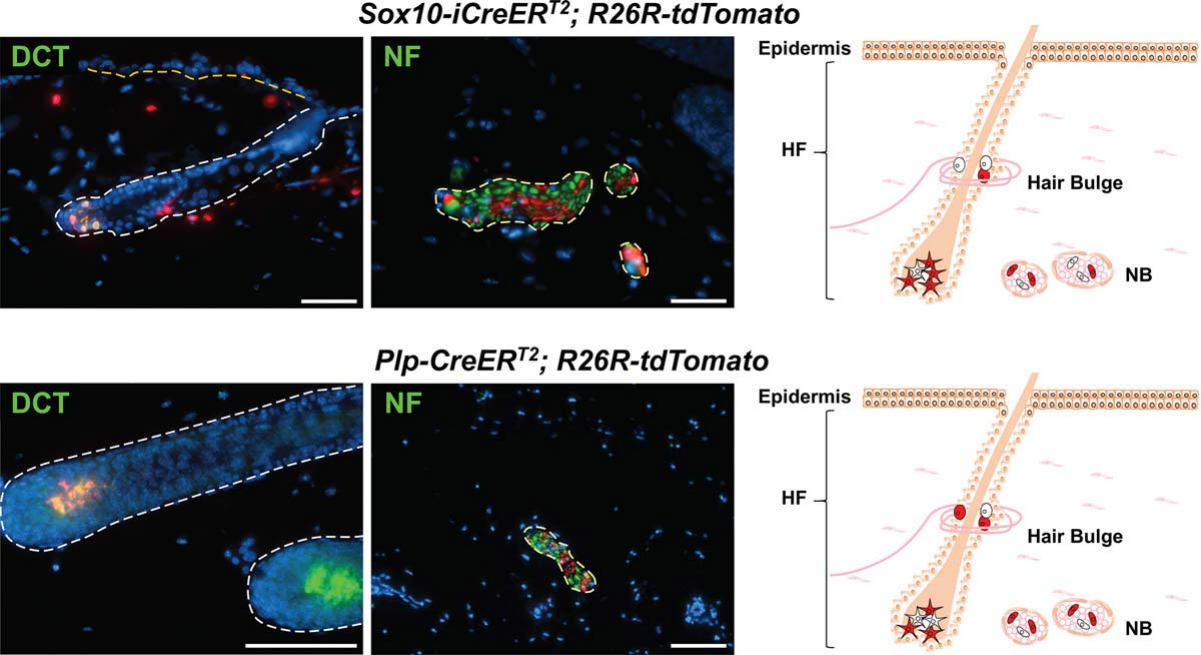
*Sox10‐iCreER^T2^* and *Plp‐CreER^T2^*‐mediated tracing of adult neural crest‐derived cells in the skin. Immunolabeling of adult skin of tamoxifen‐free animals carrying inducible *Sox10‐iCreER^T2^* (Simon et al., 2012) and *Plp‐CreER^T2^* (Leone et al., 2003), respectively, reveals leakiness of these driver lines in combination with the Cre‐reporter allele *R26R‐tdTomato* (red) in melanocyte stem cells and glial cells present in or around the hair bulge, melanocytes (DCT (green); star‐like cells in the schematics), and Schwann cells in nerve bundles (NB). NF marks axons of nerves. Hoechst nuclei counterstaining, scale bars 50 µm. Note that tamoxifen treatment of adult animals results in significantly enhanced CreER^*T2*^ activity in peripheral glia and melanocytes in both *Sox10‐iCreER^T2^* and *Plp‐CreER^T2^* lines (Parfejevs et al., 2018)

However, inducible Cre activity in the *Plp‐CreER^T2^* line turned out not to be specific for the glial lineage (Hari et al., 2012; Leone et al., 2003). Indeed, in *PLP‐CreER^T2^/R26R* embryos, TM treatment at early stages of neural crest development (E9.5) resulted in prominent labeling of peripheral neurons, glia, and melanocytes, i.e., a fate map highly reminiscent of multipotent neural crest cells (Hari et al., 2012). The expression pattern of β‐galactosidase became gradually restricted upon TM injection at later stages. However, at all stages examined, the melanocytic lineage was marked with considerable efficiency upon *PLP‐CreER^T2^*‐mediated recombination. Likewise, induction of *PLP‐CreER^T2^*‐driven recombination in adult mice marked a substantial fraction of skin melanocytes, independently of the Cre‐reporter allele used (Parfejevs et al., 2018). Some of this expression might be due to CreER^T2^ leakiness during early neural crest development, given that in adult skin of both *PLP‐CreER^T2^/R26R‐tdTomato* and *Sox10‐iCreER^T2^/R26R‐tdTomato* mice, about 25% of all hair follicles contain recombined melanocytes even in the absence of any TM treatment (Figure 2) (Parfejevs and Sommer, unpublished). However, TM injection in the adult significantly induced Cre‐reporter expression in both peripheral glia and melanocytes in the skin (Parfejevs et al., 2018), demonstrating persistent activity of *PLP‐CreER^T2^* and *Sox10‐iCreER^T2^* in these adult tissues. Thus, as with *Sox10‐CreER^T2^*‐expressing mice, different neural crest derivatives can be traced by means of the *PLP‐CreER^T2^*‐line, albeit with an apparently lower recombination efficiency. Recently, Kaucka and colleagues made use of this feature to carry out clonal analysis of cranial neural crest cells in *PLP‐CreER^T2^*/*R26R‐Confetti* embryos to confirm data obtained with a *Sox10‐iCreER^T2^* line (Kaucka et al., 2016).

In conclusion, distinct genetic mouse lines are available for fate mapping premigratory and migratory neural crest cells. Together with Cre‐driver lines specific for fate‐restricted precursor cells that we have not covered in the present review, there is an increasing tool set available to the community to study the neural crest lineage tree and the molecular mechanisms shaping it. The finding that several Cre and CreER^T2^ driver lines expected to exhibit lineage‐specific expression appear to mark multipotent neural crest cells (although with quite divergent recombination efficiencies) could simply reflect unfaithful transgene expression. Alternatively, however, migratory neural crest cells as well as, for instance, cells in peripheral nerves might comprise distinct subpopulations expressing supposedly lineage‐specific markers together with multipotency markers. Conceivably, such cell populations may be more or less ready to respond to the activity of cues controlling fate decisions during development or upon injury. To address such issues, the genetic approaches for prospective lineage tracing of neural crest cells described herein will have to be complemented with other methods, notably including retrospective lineage tracing by single cell transcriptome analysis.

## References

[dvg23105-bib-0001] Adameyko, I. , Lallemend, F. , Aquino, J. B. , Pereira, J. A. , Topilko, P. , Müller, T. , … Ernfors, P. (2009). Schwann cell precursors from nerve innervation are a cellular origin of melanocytes in skin. Cell, 139, 366–379. 19837037 10.1016/j.cell.2009.07.049

[dvg23105-bib-0002] Aoto, K. , Sandell, L. L. , Butler Tjaden, N. E. , Yuen, K. C. , Watt, K. E. N. , Black, B. L. , … Trainor, P. A. (2015). Mef2c‐F10N enhancer driven β‐galactosidase (LacZ) and Cre recombinase mice facilitate analyses of gene function and lineage fate in neural crest cells. Developmental Biology, 402, 3–16. 25794678 10.1016/j.ydbio.2015.02.022 PMC4433593

[dvg23105-bib-0003] Baggiolini, A. , Varum, S. , Mateos, J. M. , Bettosini, D. , John, N. , Bonalli, M. , … Sommer, L. (2015). Premigratory and migratory neural crest cells are multipotent in vivo. Cell Stem Cell, 16, 314–322. 25748934 10.1016/j.stem.2015.02.017

[dvg23105-bib-0004] Brault, V. , Moore, R. , Kutsch, S. , Ishibashi, M. , Rowitch, D. H. , McMahon, A. P. , … Kemler, R. (2001). Inactivation of the beta‐catenin gene by Wnt1‐Cre‐mediated deletion results in dramatic brain malformation and failure of craniofacial development. Development, 128, 1253–1264. 11262227 10.1242/dev.128.8.1253

[dvg23105-bib-0005] Breau, M. A. , Pietri, T. , Stemmler, M. P. , Thiery, J. P. , & Weston, J. A. (2008). A nonneural epithelial domain of embryonic cranial neural folds gives rise to ectomesenchyme. Proceedings of the National Academy of Sciences of the United States of America, 105, 7750–7755. 18515427 10.1073/pnas.0711344105 PMC2408482

[dvg23105-bib-0006] Bremer, M. , Fröb, F. , Kichko, T. , Reeh, P. , Tamm, E. R. , Suter, U. , & Wegner, M. (2011). Sox10 is required for Schwann‐cell homeostasis and myelin maintenance in the adult peripheral nerve. Glia, 59, 1022–1032. 21491499 10.1002/glia.21173

[dvg23105-bib-0007] Bronner, M. E. , & Simões‐Costa, M. (2016). The neural crest migrating into the twenty‐first century. Current Topics in Developmental Biology, 116, 115–134. 26970616 10.1016/bs.ctdb.2015.12.003 PMC5100668

[dvg23105-bib-0008] Büchmann‐Møller, S. , Miescher, I. , John, N. , Krishnan, J. , Deng, C.‐X. , & Sommer, L. (2009). Multiple lineage‐specific roles of Smad4 during neural crest development. Developmental Biology, 330, 329–338. 19361496 10.1016/j.ydbio.2009.04.001

[dvg23105-bib-0063] Chai, Y. , Jiang, X. , Ito, Y. , Bringas, P. , Han, J. , Rowitch, D. H. , … Sucov, H. M. (2000). Fate of the mammalian cranial neural crest during tooth and mandibular morphogenesis. Development, 127, 1671–1679. 10725243 10.1242/dev.127.8.1671

[dvg23105-bib-0009] Chen, G. , Ishan, M. , Yang, J. , Kishigami, S. , Fukuda, T. , Scott, G. , … Liu, H.‐X. (2017). Specific and spatial labeling of P0‐Cre versus Wnt1‐Cre in cranial neural crest in early mouse embryos. Genesis, 55, e23034. 10.1002/dvg.23034 PMC5473950 28371069

[dvg23105-bib-0010] Creuzet, S. , Couly, G. , Vincent, C. , & Le Douarin, N. M. (2002). Negative effect of Hox gene expression on the development of the neural crest‐derived facial skeleton. Development, 129, 4301–4313. 12183382 10.1242/dev.129.18.4301

[dvg23105-bib-0011] Danielian, P. S. , Muccino, D. , Rowitch, D. H. , Michael, S. K. , & McMahon, A. P. (1998). Modification of gene activity in mouse embryos in utero by a tamoxifen‐inducible form of Cre recombinase. Current Biology, 8, 1323–13S2. 9843687 10.1016/s0960-9822(07)00562-3

[dvg23105-bib-0012] Delmas, V. , Martinozzi, S. , Bourgeois, Y. , Holzenberger, M. , & Larue, L. (2003). Cre‐mediated recombination in the skin melanocyte lineage. Genesis (New York, N.Y.: 2000), 36, 73–80. 10.1002/gene.10197 12820167

[dvg23105-bib-0013] Deo, M. , Huang, J. L.‐Y. , Fuchs, H. , de Angelis, M. H. , & Van Raamsdonk, C. D. (2013). Differential effects of neurofibromin gene dosage on melanocyte development. The Journal of Investigative Dermatology, 133, 49–58. 22810304 10.1038/jid.2012.240

[dvg23105-bib-0014] Doerflinger, N. H. , Macklin, W. B. , & Popko, B. (2003). Inducible site‐specific recombination in myelinating cells. Genesis (New York, N.Y.: 2000), 35, 63–72. 10.1002/gene.10154 12481300

[dvg23105-bib-0015] Dyachuk, V. , Furlan, A. , Shahidi, M. K. , Giovenco, M. , Kaukua, N. , Konstantinidou, C. , … Adameyko, I. (2014). Parasympathetic neurons originate from nerve‐associated peripheral glial progenitors. Science, 345, 82–87. 24925909 10.1126/science.1253281

[dvg23105-bib-0016] Dymecki, S. M. , & Tomasiewicz, H. (1998). Using Flp‐recombinase to characterize expansion of Wnt1‐expressing neural progenitors in the mouse. Developmental Biology, 201, 57–65. 9733573 10.1006/dbio.1998.8971

[dvg23105-bib-0017] Engleka, K. A. , Manderfield, L. J. , Brust, R. D. , Li, L. , Cohen, A. , Dymecki, S. M. , & Epstein, J. A. (2012). Islet1 derivatives in the heart are of both neural crest and second heart field origin. Circulation Research, 110, 922–926. 22394517 10.1161/CIRCRESAHA.112.266510 PMC3355870

[dvg23105-bib-0018] Espinosa‐Medina, I. , Outin, E. , Picard, C. A. , Chettouh, Z. , Dymecki, S. , … Brunet, J.‐F. (2014). Neurodevelopment. Parasympathetic ganglia derive from Schwann cell precursors. Science, 345, 87–90. 24925912 10.1126/science.1253286

[dvg23105-bib-0019] Feil, R. , Brocard, J. , Mascrez, B. , LeMeur, M. , Metzger, D. , & Chambon, P. (1996). Ligand‐activated site‐specific recombination in mice. Proceedings of the National Academy of Sciences of the United States of AmericaU S A, 93, 10887–10890. 10.1073/pnas.93.20.10887 PMC38252 8855277

[dvg23105-bib-0020] Feil, R. , Wagner, J. , Metzger, D. , & Chambon, P. (1997). Regulation of Cre recombinase activity by mutated estrogen receptor ligand‐binding domains. Biochemical and Biophysical Research Communications, 237, 752–757. 9299439 10.1006/bbrc.1997.7124

[dvg23105-bib-0021] Furlan, A. , Dyachuk, V. , Kastriti, M. E. , Calvo‐Enrique, L. , Abdo, H. , Hadjab, S. , … Adameyko, I. (2017). Multipotent peripheral glial cells generate neuroendocrine cells of the adrenal medulla. Science, 357, eaal3753. 28684471 10.1126/science.aal3753 PMC6013038

[dvg23105-bib-0022] Hari, L. , Brault, V. , Kléber, M. , Lee, H. Y. , Ille, F. , Leimeroth, R. , … Sommer, L. (2002). Lineage‐specific requirements of β‐catenin in neural crest development. The Journal of Cell Biology, 159, 867–880. 12473692 10.1083/jcb.200209039 PMC2173383

[dvg23105-bib-0023] Hari, L. , Miescher, I. , Shakhova, O. , Suter, U. , Chin, L. , Taketo, M. , … Sommer, L. (2012). Temporal control of neural crest lineage generation by Wnt/β‐catenin signaling. Development, 139, 2107–2117. 22573620 10.1242/dev.073064

[dvg23105-bib-0024] Hatzistergos, K. E. , Takeuchi, L. M. , Saur, D. , Seidler, B. , Dymecki, S. M. , Mai, J. J. , … Hare, J. M. (2015). cKit + cardiac progenitors of neural crest origin. Proceedings of the National Academy of Sciences, 112, 13051–13056. 10.1073/pnas.1517201112 PMC4620867 26438843

[dvg23105-bib-0025] He, F. , & Soriano, P. (2015). Sox10ER(T2) CreER(T2) mice enable tracing of distinct neural crest cell populations. Developmental Dynamics, 244, 1394–1403. 26250625 10.1002/dvdy.24320 PMC4619116

[dvg23105-bib-0026] Hunter, N. L. , Awatramani, R. B. , Farley, F. W. , & Dymecki, S. M. (2005). Ligand‐activated Flpe for temporally regulated gene modifications. Genesis (New York, N.Y. : 2000), 41, 99–109. 10.1002/gene.20101 15729687

[dvg23105-bib-0027] Jarad, G. , & Miner, J. H. (2009). The Pax3‐Cre transgene exhibits a rostrocaudal gradient of expression in the skeletal muscle lineage. Genesis, 47, 1–6. 18942111 10.1002/dvg.20447 PMC2759890

[dvg23105-bib-0028] Jensen, P. , Farago, A. F. , Awatramani, R. B. , Scott, M. M. , Deneris, E. S. , & Dymecki, S. M. (2008). Redefining the serotonergic system by genetic lineage. Nature Neuroscience, 11, 417–419. 18344997 10.1038/nn2050 PMC2897136

[dvg23105-bib-0029] Jia, Q. , McDill, B. W. , Li, S. Z. , Deng, C. , Chang, C. P. , & Chen, F. (2007). Smad signaling in the neural crest regulates cardiac outflow tract remodeling through cell autonomous and non‐cell autonomous effects. Developmental Biology, 311, 172–184. 17916348 10.1016/j.ydbio.2007.08.044 PMC2692609

[dvg23105-bib-0030] Jiang, X. , Rowitch, D. H. , Soriano, P. , McMahon, A. P. , & Sucov, H. M. (2000). Fate of the mammalian cardiac neural crest. Development, 127, 1607–1616. 10725237 10.1242/dev.127.8.1607

[dvg23105-bib-0064] Kaucka, M. , Ivashkin, E. , Gyllborg, D. , Zikmund, T. , Tesarova, M. , Kaiser, J. , … Adameyko, I. (2016). Analysis of neural crest‐derived clones reveals novel aspects of facial development. Science Advances, 2, e1600060–e1600060. 27493992 10.1126/sciadv.1600060 PMC4972470

[dvg23105-bib-0031] Kaukua, N. , Shahidi, M. K. , Konstantinidou, C. , Dyachuk, V. , Kaucka, M. , Furlan, A. , … Adameyko, I. (2014). Glial origin of mesenchymal stem cells in a tooth model system. Nature, 513, 551–554. 25079316 10.1038/nature13536

[dvg23105-bib-0032] Kléber, M. , Lee, H.‐Y. , Wurdak, H. , Buchstaller, J. , Riccomagno, M. M. , Ittner, L. M. , … Sommer, L. (2005). Neural crest stem cell maintenance by combinatorial Wnt and BMP signaling. The Journal of Cell Biology, 169, 309–320. 15837799 10.1083/jcb.200411095 PMC2171862

[dvg23105-bib-0033] Kuhlbrodt, K. , Herbarth, B. , Sock, E. , Hermans‐Borgmeyer, I. , & Wegner, M. (1998). Sox10, a novel transcriptional modulator in glial cells. The Journal of Neuroscience : The Official Journal of the Society for Neuroscience, 18, 237–250. 9412504 10.1523/JNEUROSCI.18-01-00237.1998 PMC6793382

[dvg23105-bib-0034] Lang, D. , Chen, F. , Milewski, R. , Li, J. , Lu, M. M. , & Epstein, J. A. (2000). Pax3 is required for enteric ganglia formation and functions with Sox10 to modulate expression of c‐ret. Journal of Clinical Investigation, 106, 963–971. 11032856 10.1172/JCI10828 PMC314346

[dvg23105-bib-0035] Laranjeira, C. , Sandgren, K. , Kessaris, N. , Richardson, W. , Potocnik, A. , Vanden Berghe, P. , & Pachnis, V. (2011). Glial cells in the mouse enteric nervous system can undergo neurogenesis in response to injury. Journal of Clinical Investigation, 121, 3412–3424. 21865647 10.1172/JCI58200 PMC3163972

[dvg23105-bib-0036] Le Douarin, N. M. , & Dupin, E. (2003). Multipotentiality of the neural crest. Current Opinion in Genetics &Amp; Development, 13, 529–536. 10.1016/j.gde.2003.08.002 14550420

[dvg23105-bib-0037] Leone, D. P. , Genoud, S. T. , Atanasoski, S. , Grausenburger, R. , Berger, P. , Metzger, D. , … Suter, U. (2003). Tamoxifen‐inducible glia‐specific Cre mice for somatic mutagenesis in oligodendrocytes and Schwann cells. Molecular and Cellular Neurosciences, 22, 430–440. 12727441 10.1016/s1044-7431(03)00029-0

[dvg23105-bib-0038] Lewis, A. E. , Vasudevan, H. N. , O'neill, A. K. , Soriano, P. , & Bush, J. O. (2013). The widely used Wnt1‐Cre transgene causes developmental phenotypes by ectopic activation of Wnt signaling. Developmental Biology, 379, 229–234. 23648512 10.1016/j.ydbio.2013.04.026 PMC3804302

[dvg23105-bib-0039] Li, J. , Chen, F. , & Epstein, J. A. (2000). Neural crest expression of Cre recombinase directed by the proximal Pax3 promoter in transgenic mice. Genesis (New York, N.Y. : 2000), 26, 162–164. 10.1002/(sici)1526-968x(200002)26:2<162::aid-gene21>3.0.co;2-r 10686619

[dvg23105-bib-0040] Liu, S. , Liu, F. , Schneider, A. E. , St, A. , T., Epstein, J. A. , & Gutstein, D. E. (2006). Distinct cardiac malformations caused by absence of connexin 43 in the neural crest and in the non‐crest neural tube. Development, 133, 2063–2073. 16624854 10.1242/dev.02374

[dvg23105-bib-0041] Macatee, T. L. , Hammond, B. P. , Arenkiel, B. R. , Francis, L. , Frank, D. U. , & Moon, A. M. (2003). Ablation of specific expression domains reveals discrete functions of ectoderm‐ and endoderm‐derived FGF8 during cardiovascular and pharyngeal development. Development, 130, 6361–6374. 14623825 10.1242/dev.00850 PMC1876660

[dvg23105-bib-0042] Matsuoka, T. , Ahlberg, P. E. , Kessaris, N. , Iannarelli, P. , Dennehy, U. , Richardson, W. D. , … Koentges, G. (2005). Neural crest origins of the neck and shoulder. Nature, 436, 347–355. 16034409 10.1038/nature03837 PMC1352163

[dvg23105-bib-0043] McKenzie, I. A. , Ohayon, D. , Li, H. , De Faria, J. P. , Emery, B. , Tohyama, K. , & Richardson, W. D. (2014). Motor skill learning requires active central myelination. Science, 346, 318–322. 25324381 10.1126/science.1254960 PMC6324726

[dvg23105-bib-0044] Nagoshi, N. , Shibata, S. , Kubota, Y. , Nakamura, M. , Nagai, Y. , Satoh, E. , … Okano, H. (2008). Ontogeny and multipotency of neural crest‐derived stem cells in mouse bone marrow, dorsal Root Ganglia, and Whisker Pad. Cell Stem Cell, 2, 392–403. 18397758 10.1016/j.stem.2008.03.005

[dvg23105-bib-0045] Parfejevs, V. , Debbache, J. , Shakhova, O. , Schaefer, S. M. , Glausch, M. , Wegner, M. , … Sommer, L. (2018). Injury‐activated glial cells promote wound healing of the adult skin in mice. Nature Communications, 9, 236. 10.1038/s41467-017-01488-2 PMC5770460 29339718

[dvg23105-bib-0046] Petersen, J. , & Adameyko, I. (2017). Nerve‐associated neural crest: peripheral glial cells generate multiple fates in the body. Current Opinion in Genetics & Development, 45, 10–14. 28242477 10.1016/j.gde.2017.02.006

[dvg23105-bib-0047] Pietri, T. , Eder, O. , Blanche, M. , Thiery, J. P. , & Dufour, S. (2003). The human tissue plasminogen activator‐Cre mouse: A new tool for targeting specifically neural crest cells and their derivatives in vivo. Developmental Biology, 259, 176–187. 12812797 10.1016/s0012-1606(03)00175-1

[dvg23105-bib-0048] Rabadán, M. A. , Herrera, A. , Fanlo, L. , Usieto, S. , Carmona‐Fontaine, C. , Barriga, E. H. , … Martí, E. (2016). Delamination of neural crest cells requires transient and reversible Wnt inhibition mediated by Dact1/2. Development, 143, 2194–2205. 27122165 10.1242/dev.134981 PMC4920176

[dvg23105-bib-0049] Shakhova, O. , & Sommer, L. (2010). *Neural crest‐derived stem cells* *StemBook*, 1–20. 10.3824/stembook.1.51.1 20614636

[dvg23105-bib-0050] Shakhova, O. , Zingg, D. , Schaefer, S. M. , Hari, L. , Civenni, G. , Blunschi, J. , … Sommer, L. (2012). Sox10 promotes the formation and maintenance of giant congenital naevi and melanoma. Nature Cell Biology, 14, 882–890. 22772081 10.1038/ncb2535

[dvg23105-bib-0051] Simon, C. , Lickert, H. , Götz, M. , & Dimou, L. (2012). Sox10‐iCreERT2 : a mouse line to inducibly trace the neural crest and oligodendrocyte lineage. genesis, 50, 506–515. 22173870 10.1002/dvg.22003

[dvg23105-bib-0052] Snippert, H. J. , van der Flier, L. G. , Sato, T. , van Es, J. H. , van den Born, M. , Kroon‐Veenboer, C. , … Clevers, H. (2010). Intestinal crypt homeostasis results from neutral competition between symmetrically dividing Lgr5 stem cells. Cell, 143, 134–144. 20887898 10.1016/j.cell.2010.09.016

[dvg23105-bib-0065] Soriano, P. (1999). Generalized lacZ expression with the ROSA26 Cre reporter strain. Nature Genetics, 21, 70–71. 9916792 10.1038/5007

[dvg23105-bib-0053] Tonks, I. D. , Nurcombe, V. , Paterson, C. , Zournazi, A. , Prather, C. , Mould, A. W. , & Kay, G. F. (2003). Tyrosinase‐Cre mice for tissue‐specific gene ablation in neural crest and neuroepithelial‐derived tissues. Genesis, 37, 131–138. 14595836 10.1002/gene.10242

[dvg23105-bib-0054] Ueharu, H. , Yoshida, S. , Kikkawa, T. , Kanno, N. , Higuchi, M. , Kato, T. , … Kato, Y. (2017). Gene tracing analysis reveals the contribution of neural crest‐derived cells in pituitary development. Journal of Anatomy, 230, 373–380. 28026856 10.1111/joa.12572 PMC5314385

[dvg23105-bib-0055] Uesaka, T. , Nagashimada, M. , & Enomoto, H. (2015). Neuronal differentiation in Schwann cell lineage underlies postnatal neurogenesis in the enteric nervous system. The Journal of Neuroscience, 35, 9879–9888. 26156989 10.1523/JNEUROSCI.1239-15.2015 PMC6605410

[dvg23105-bib-0056] Wang, X. , & Astrof, S. (2016). Neural crest cell‐autonomous roles of fibronectin in cardiovascular development. Development, 143, 88–100. 26552887 10.1242/dev.125286 PMC4725203

[dvg23105-bib-0057] Wislet‐Gendebien, S. , Laudet, E. , Neirinckx, V. , Alix, P. , Leprince, P. , Glejzer, A. , … Rogister, B. (2012). Mesenchymal stem cells and neural crest stem cells from adult bone marrow: characterization of their surprising similarities and differences. Cellular and Molecular Life Sciences, 69, 2593–2608. 22349262 10.1007/s00018-012-0937-1 PMC11114712

[dvg23105-bib-0058] Wong, C. E. , Paratore, C. , Dours‐Zimmermann, M. T. , Rochat, A. , Pietri, T. , Suter, U. , … Sommer, L. (2006). Neural crest‐derived cells with stem cell features can be traced back to multiple lineages in the adult skin. The Journal of Cell Biology, 175, 1005–1015. 17158956 10.1083/jcb.200606062 PMC2064709

[dvg23105-bib-0059] Woodworth, M. B. , Girskis, K. M. , & Walsh, C. A. (2017). Building a lineage from single cells: genetic techniques for cell lineage tracking. Nature Reviews Genetics, 18, 230–244. 10.1038/nrg.2016.159 PMC5459401 28111472

[dvg23105-bib-0060] Yamauchi, Y. , Abe, K. , Mantani, A. , Hitoshi, Y. , Suzuki, M. , Osuzu, F. , … Yamamura, K‐I. (1999). A novel transgenic technique that allows specific marking of the neural crest cell lineage in mice. Developmental Biology, 212, 191–203. 10419695 10.1006/dbio.1999.9323

[dvg23105-bib-0061] Zervas, M. , Millet, S. , Ahn, S. , & Joyner, A. L. (2004). Cell behaviors and genetic lineages of the mesencephalon and rhombomere 1. Neuron, 43, 345–357. 15294143 10.1016/j.neuron.2004.07.010

[dvg23105-bib-0062] Zurkirchen, L. , & Sommer, L. (2017). Quo vadis: tracing the fate of neural crest cells. Current Opinion in Neurobiology, 47, 16–23. 28753439 10.1016/j.conb.2017.07.001

